# The Iliotibial Band: A Complex Structure with Versatile Functions

**DOI:** 10.1007/s40279-021-01634-3

**Published:** 2022-01-24

**Authors:** L. A. Hutchinson, G. A. Lichtwark, R. W. Willy, L. A. Kelly

**Affiliations:** 1 School of Human Movement and Nutrition, The University of Queensland, Brisbane, QLD Australia; 2 School of Physical Therapy and Rehabilitation Science, University of Montana, Missoula, MT USA

## Abstract

The development of a pronounced iliotibial band (ITB) is an anatomically distinct evolution of humans. The mechanical behaviour of this “new” structure is still poorly understood and hotly debated in current literature. Iliotibial band syndrome (ITBS) is one of the leading causes of lateral knee pain injuries in runners. We currently lack a comprehensive understanding of the healthy behaviour of the ITB, and this is necessary prior to further investigating the aetiology of pathologies like ITBS. Therefore, the purpose of this narrative review was to collate the anatomical, biomechanical and clinical literature to understand how the mechanical function of the ITB is influenced by anatomical variation, posture and muscle activation. The complexity of understanding the mechanical function of the ITB is due, in part, to the presence of its two in-series muscles: gluteus maximus (GMAX) and tensor fascia latae (TFL). At present, we lack a fundamental understanding of how GMAX and TFL transmit force through the ITB and what mechanical role the ITB plays for movements like walking or running. While there is a range of proposed ITBS treatment strategies, robust evidence for effective treatments is still lacking. Interventions that directly target the running biomechanics suspected to increase either ITB strain or compression of lateral knee structures may have promise, but clinical randomised controlled trials are still required.

## Key Points

**Table:** 

The iliotibial band has five commonly cited distal insertion points, all of which have the ability to transmit significant force and thus contribute to its potential mechanical functions
The complexity of iliotibial band syndrome and its relationship with the in-series musculature is poorly understood and should be further researched prospectively to determine the true aetiology of iliotibial band syndrome

## Introduction

**Fig. 1:**
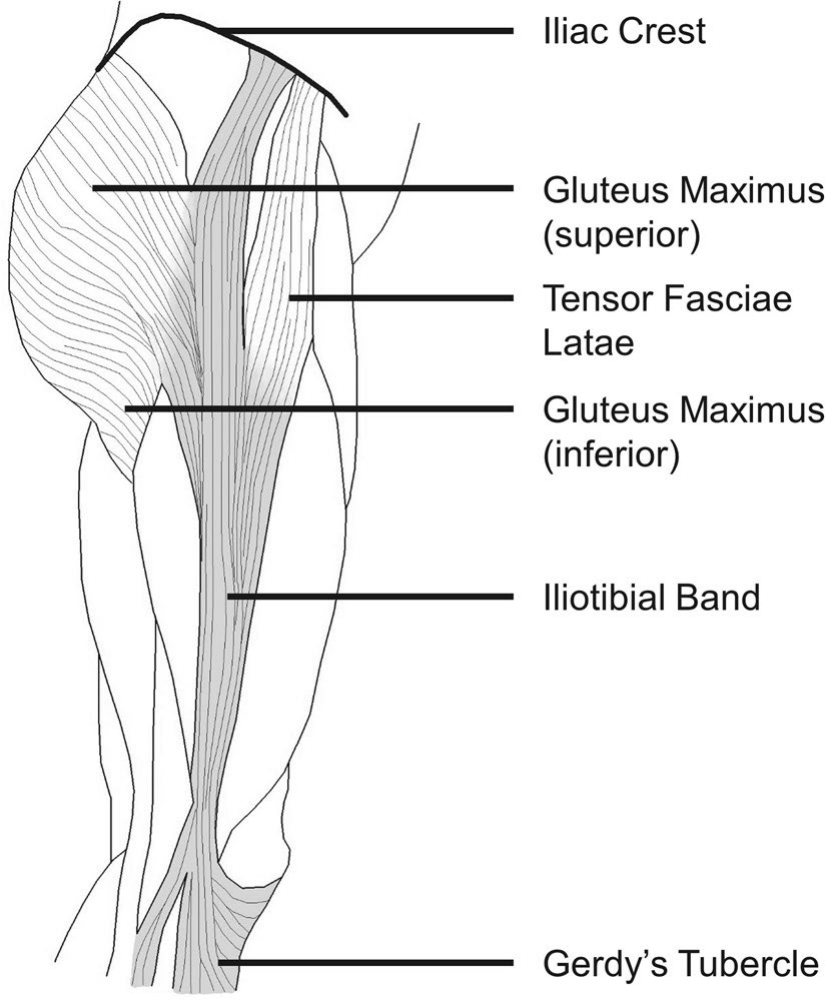
Traditionally taught anatomy of the iliotibial band; originating on the iliac crest between the gluteus medius and sartorius muscles (not pictured for clarity) and inserting distally at Gerdy’s tubercle on the lateral tibia. Two in-series muscles (gluteus maximus and tensor fasciae latae) insert partially and fully, respectively, into the iliotibial band

The iliotibial band (ITB) is a tough, fibrous fascial tissue that spans from the iliac crest to the lateral proximal tibia, and in its current evolutionary form has been associated with the erect posture of humans (Fig. 1) [1–4]. The various functional roles of the ITB seem to be dependent on posture, and thus activity choice [5–7]. This may be due to the presence of two in-series muscles: the gluteus maximus (GMAX) and the tensor fasciae latae (TFL), as well as the anatomical path of the ITB crossing both the hip and knee joints [8]. Simplified models, invasive methods, cadaveric work and simple static investigations have all contributed to explain the function of a healthy ITB. However, the precise mechanical function and indeed, even the basic anatomy of the ITB, is still poorly understood [5, 7–13]. The ITB is thought to function as a strut during walking, acting as both a hip and a knee stabiliser, primarily in the frontal plane [1, 14–21]. It has also been suggested that it may store considerable magnitudes of elastic energy during walking [1, 8, 16].

The mechanical importance of the ITB is highlighted by the prevalence with which it is injured, particularly in runners. ITB pain is common in runners (a 5–14% prevalence of all running-related injuries) [22]. We would expect that such a common injury would be well documented, with reliable options for diagnosis and treatment; unfortunately the reality is quite the opposite. Some recent reviews have been quite critical of the diagnostic and treatment strategies for ITBS [23, 24]. A first example is the shift from classification of ITBS as a friction syndrome [25] to that of a compression syndrome [26, 27] or impingement model [28] (described in Sect. 6.1). Authors have also suggested that ITBS could be a function of reduced hip muscular strength [29], while others present evidence to challenge this theory [24, 27, 30]. Current clinical understanding of ITBS is lacking, highlighting the need to understand the mechanical function of the ITB to better inform clinical practice.

## Review Purpose

The purpose of this literature review was to collate anatomical and biomechanical information that informs our knowledge of the mechanical function of the ITB, to better understand the aetiology, clinical examination and treatment of ITBS. This critical narrative review specifically focuses on the factors influencing strain and tension in the ITB, with a focus on the roles of the in-series musculature. Understanding how the ITB is tensioned during different phases of the gait cycle, how this is influenced by anatomical variation, and what mechanical function this might play, is critical for understanding the aetiology, clinical presentation and treatment of individuals with ITBS.

## Anatomical Variance

### Evolutionary Uniqueness

Compared to non-human primates, the GMAX muscle in humans is much larger in size, providing a role in enhancing trunk stabilisation [31–33]. Stern Jr. suggests that humans evolved an “entirely new” insertion point of GMAX into the overlying fascia, which sparked interest into the identification and examination of the functions that accompany this “new” anatomical configuration [33]. In fact, this “new” insertion point, or the development of a pronounced ITB, is unique to humans, and is anatomically distinct from the fascia lata of other primates [1]. When we consider that there are apparently no mammalian animals lacking a TFL [1], the uniqueness of the presence of the ITB in humans is fascinating. In all other animals, the TFL terminates in the superior thigh inserting into the femur near the greater trochanter [1]. Thus, bipediality in humans has been considered to be spurred by the development of a large GMAX, the change in position of the pelvis from horizontal to vertical, and the formation of an ITB [1]. In support of this theory, it is interesting to note that humans are not born with a distinct, distally inserting ITB, but rather this band is formed later after we begin to walk bipedally [1].

### Muscular Contributions

**Fig. 2:**
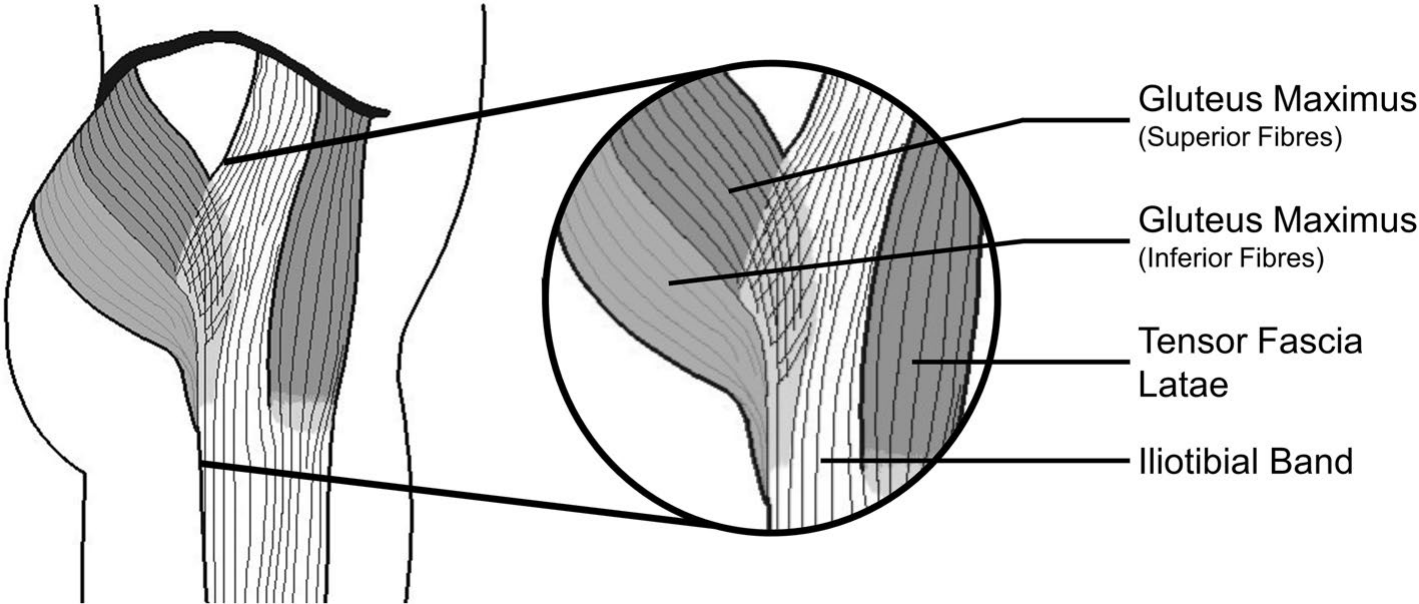
Iliotibial band anatomy showing insertion of in-series muscles (gluteus maximus and tensor fascia latae)

The anatomy of the ITB also extends to the anatomy of the in-series musculature: GMAX and TFL muscles. These muscles directly insert (either partially or fully) into the ITB, contributing to the functional mechanics of the ITB (Fig. 2). Generally, the TFL pulls anterosuperiorly on the ITB to flex the hip, while the GMAX pulls posteriorly to extend the hip [1, 4, 16, 33]. Discrepancies in the descriptions of muscle attachment and distal insertion sites of the ITB, as well as the variances in ITB size between subjects, factor into our lack of understanding of the function of the ITB and its anatomy. Specifically, the literature has more debates regarding the anatomical variances of GMAX than TFL.

Historically, the insertion of GMAX into the ITB has been poorly described [1, 3, 16, 34, 35]. The literature often fails to acknowledge that GMAX has two distinct portions: the superior portion inserts into the ITB [3] and the inferior portion inserts into the femur [1, 4, 35]. The superior portion of GMAX was initially described in 1948 as being 75% of GMAX, seemingly with no reported experimental evidence [34]. More recently, GMAX’s insertion into the ITB was quantified by dissecting cadavers and identifying that this superior, superficial portion ranges from ~ 40–70% of GMAX’s total mass [8]. Despite the reported variability in the proportion of GMAX inserting into the ITB, it is substantial enough that the ITB be considered to behave as an insertional tendon of GMAX [36]. The inferior GMAX fibres form an ascending tendon that inserts directly into the femur at the linea aspera [1, 8, 33]. The femoral insertion tendon (or tendinous tissue) is short, and considered to transmit less force than the portion inserting into the ITB [4, 36]. The differing insertion points, and thus force transmission pathways of these two portions of GMAX, undoubtedly contribute to differing functions (see Sect. 4.4). The ITB’s relative size varies substantially across the population, with standard deviations in thickness and width measures ranging from 19 to 68% of the average thickness and width [8, 37]. These variations in ITB size could partially be explained by the substantial variability in the percentage of GMAX muscle insertion into the ITB, which likely impacts the forces experienced by the ITB and energetic contribution to movement [8].

Generally, fascia is often hypothesized to broaden the insertion of muscles by distributing or redirecting muscle force transmission [38–40]. Given that the ITB broadens the insertion of GMAX muscle and facilitates the insertion of TFL muscle, it can be ascertained that the ITB plays a role in the transmission of forces from these two muscles across both the knee and the hip. The specific function(s) of the ITB and the influence of variations in activation levels of the in-series muscles remain a relative mystery.

### Distal Insertion

**Fig. 3:**
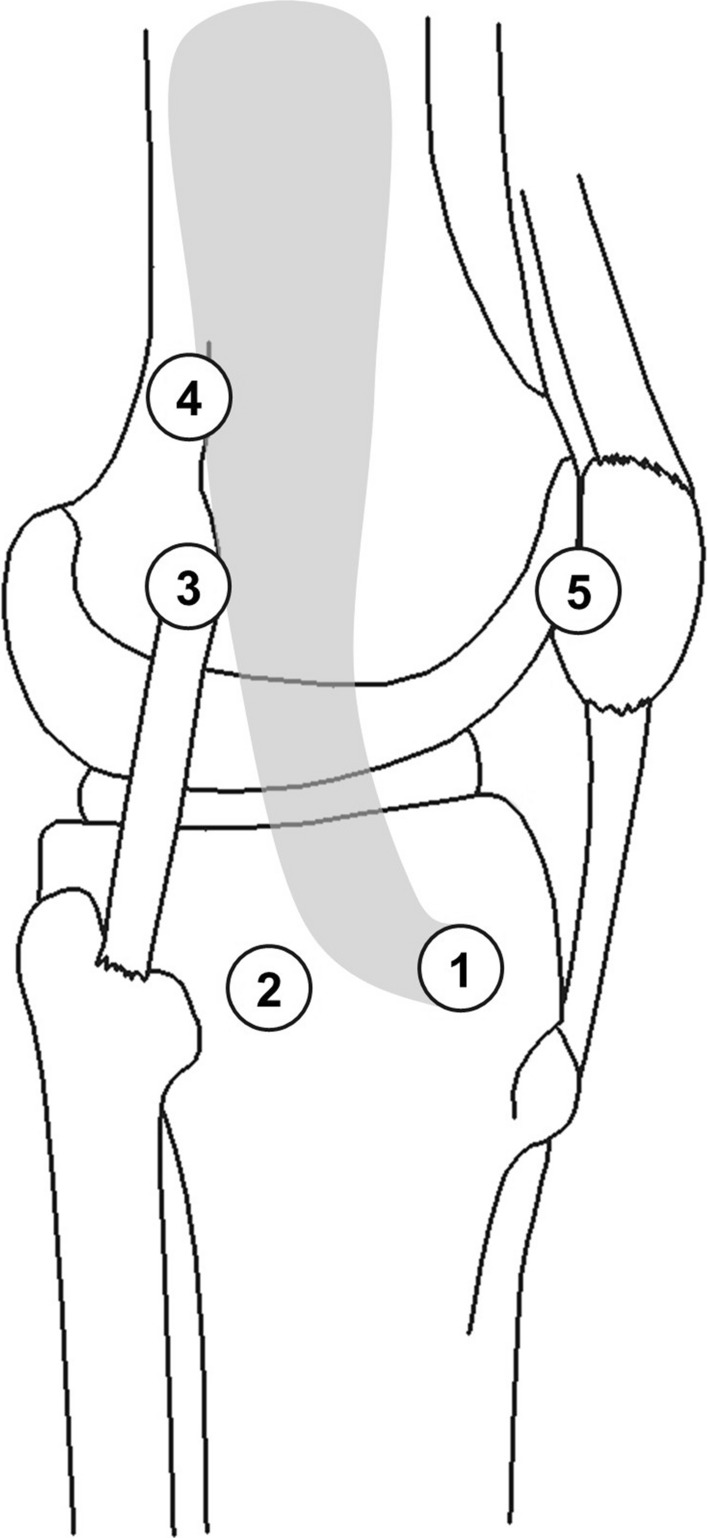
Various possible distal insertions of the iliotibial band have been reported in the literature: (1) Gerdy’s tubercle [1–4, 15, 18–21, 41], (2) the insertion point of the lateral femorotibial ligament [21, 41], (3) supracondylar femur (Kaplan’s distal fibers) [1, 18, 21, 41], (4) along the linea aspera (Kaplan’s proximal fibers) [5, 18, 21, 41], and (5) the patella [15, 18–21, 41]

While the distal insertion of the ITB is what makes it unique to humans, descriptions of this insertion vary widely [1, 16]. Though the ITB’s insertion at Gerdy’s tubercle is unanimous across all authors [1–4, 14, 15, 17–21, 41], there has been substantial work investigating other claims for distal attachment (summarized in Fig. 3). Four other commonly published distal insertion points of the ITB are: a shared insertion with the femoral tibial ligament [21, 41], the supracondylar femur shared with the lateral collateral ligament (Kaplan’s distal fibers) [1, 18, 21, 41], along the linea aspera (Kaplan’s proximal fibers) [1, 5, 18, 21, 41], and to the patella [15, 18–21, 41]. Godin et al.’s recent (2017) work quantified these attachment sites and also defined the two discrete Kaplan fiber bundles [41]. These bundles were originally described by Kaplan (1958) as a singular band of fibres attaching to the upper portion of the lateral femoral condyle [1].

Recent literature has argued that the ITB should not be considered a discrete structure because it is merely a thickening of the fascia lata with margins that cannot clearly be distinguished during cadaveric dissections [26, 36]. The lack of clearly distinguishable borders is increasingly evident when examining the discrepancies among claims of distal insertions. However, perhaps these differences may be attributed to limited tension in the ITB of dissected cadavers, making the ITB’s borders hard to distinguish, compared to surface anatomy descriptions where the ITB’s borders are visible through the skin [5, 26]. Varying descriptions in the literature of distal insertions of the ITB complicate a greater understanding of the function of the ITB. The substantial variability in the descriptions of distal insertion of the ITB may suggest that the ITB is not a discrete structure [26, 36]. Alternatively, the different insertions may represent discrete force transmission pathways through the ITB, which are representative of the large number of potential mechanical functions of the ITB that are dependent on posture and muscular activation.

## Mechanical Function

The mechanical behaviour of the ITB is quite literally tethered to the in-series muscles that insert directly into it (TFL and GMAX). There is an abundance of literature suggesting the independent function of these muscles; however, much of the literature is conflicting or contradictory, leaving room to explore the potential combined function of these muscles through the ITB.

### Tensor Facia Latae

**Fig. 4:**
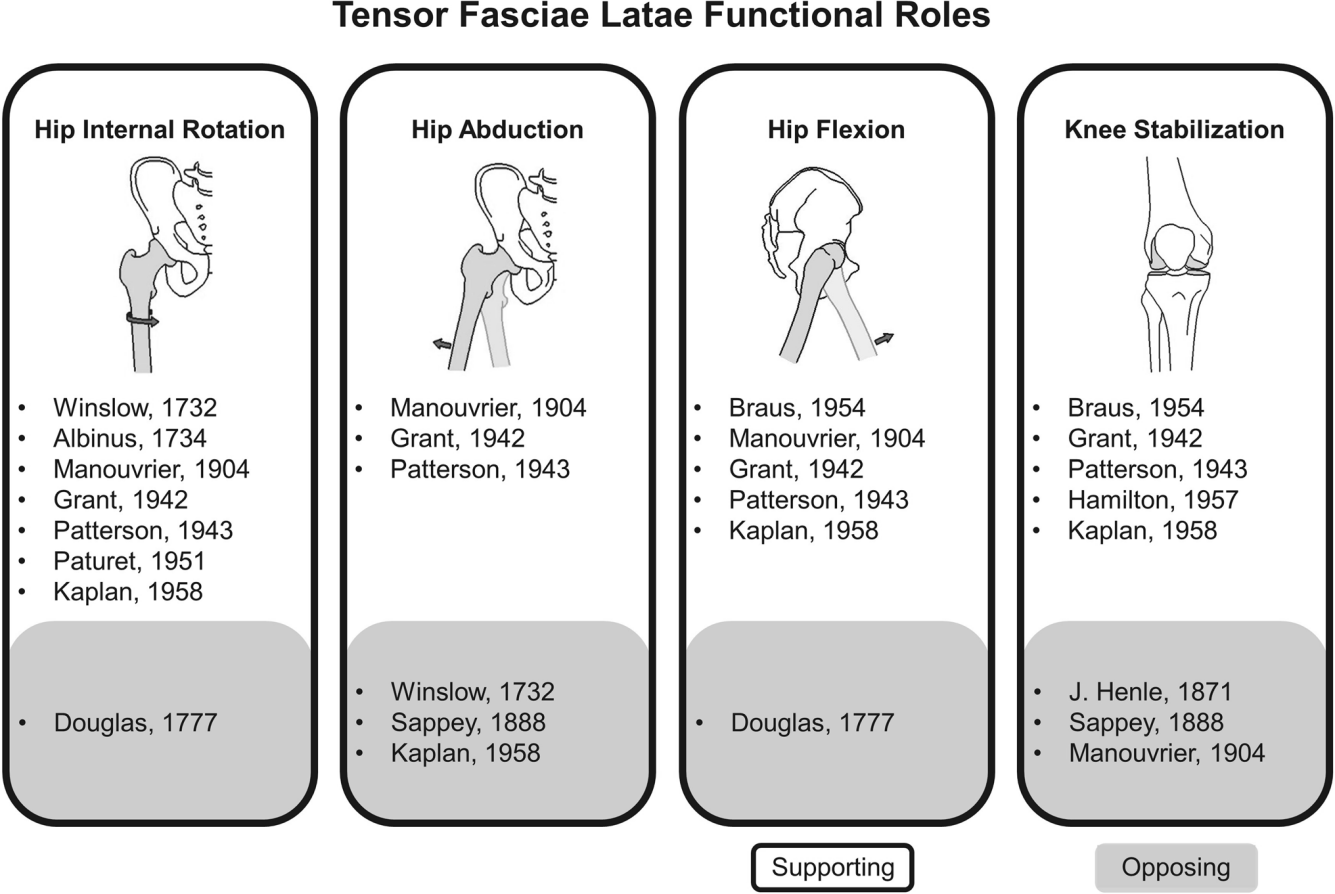
Summary of the diversity of findings regarding the function of the tensor fasciae latae (citations and summary generated from Kaplan [1]). The citations in the white background support the functional claim while the citations in grey oppose or contradict it

The mechanical function of the TFL has confounded researchers, with little agreement in the literature (Kaplan’s summary is visualized in Fig. 4) [1]. The consensus regarding the function of TFL suggests it contributes to hip internal rotation, hip flexion and stabilization of the knee. TFL’s role in hip abduction is more contested [1]; however, its high electromyographic (EMG) activity in isolated abduction has led some to conclude that hip abduction is a primary function of TFL [4, 16]. The TFL was considered to be “the sixth muscle of the tibia” in earlier literature, alluding to its hypothesized role at the knee joint [1, 42]. It follows that for TFL to apply any force at the knee, it would be transmitted via the ITB. The current consensus in the literature does not support an active contribution to knee joint movement, but rather considers the TFL (through the ITB) to play a stabilization role at the knee [1]. The role of the ITB in knee stabilisation is discussed in detail below (see Sect. 4.3).

Gottschalk et al. [16] hypothesized that during normal walking, the ITB functions as a “strut” providing frontal plane stabilization of the hip. More recently, Neumann [43] investigated this hypothesis through the theoretical potential actions of the muscles of the hip, and concluded that given TFL’s frontal plane moment arm it could function to stabilise the pelvis in the frontal plane. Due to its biarticular nature, and because it shares an insertion onto the ITB with a muscle that opposes hip flexion (GMAX), the mechanical role of TFL likely depends on the posture of the hip and knee when force is produced – in particular, the interaction with other agonists and antagonists, most notably GMAX.

### Gluteus Maximus

GMAX is considered a primary extensor muscle of the hip joint because of its large muscle volume and large hip extension moment arm in the sagittal plane, which positions it to be a major force producing muscle of the lower limb [43]. GMAX assists in external hip joint rotation, hip abduction and, importantly, tensioning of the ITB [4, 43–45]. Given its large physiological cross-sectional area [4, 31], large hip extension moment arm, and high proportion of fibres that insert onto the ITB [8], GMAX is likely able to transmit greater force through the ITB than the TFL in the sagittal plane. The biomechanical role of the portion of GMAX muscle that inserts on the ITB is still a matter of considerable debate.

**Fig. 5:**
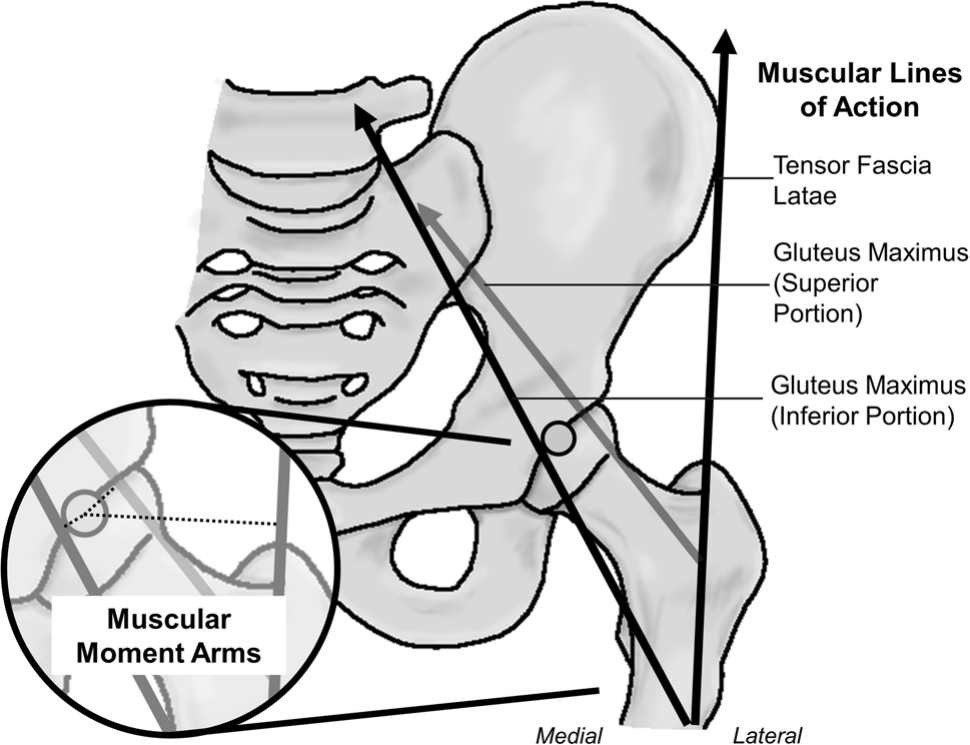
Sagittal view of the hip joint using muscular lines of action from Neumann [43]. The dark black muscular lines of action for TFL and GMAX inferior are as originally published [43], while the light grey GMAX superior portion is newly presented here to support the EMG evidence from Selkowitz et al. [45] to allow for GMAX superior to abduct the hip. Bottom left: the predicted muscular corresponding moment arms (dashed lines)

The work by Neumann [43] investigated the theoretical potential of the hip musculature to function at the hip based on muscular lines of action and moment arms. This work supports the prior claims for GMAX to perform primary actions of hip extension and external rotation [4, 44, 45]. However, Neumann’s [43] straight-line, uniarticular models of muscle action are based on cadaveric work from Dostal and Andrews [46] and model GMAX as only the portion that inserts into the femur. When we consider the superior portion of GMAX that inserts more proximally into the ITB, GMAX may also have an abduction moment arm about the hip joint (Fig. 5), and therefore have a role in generating hip abduction moments. This assertion is supported by electromyography data showing significant activation of the superior portion of the GMAX during hip abduction tasks [45].

Understanding the role of the superior portion of the GMAX (i.e., the portion inserting into the ITB) at the hip is important for determining when force is transmitted through the ITB. For instance, during the early stance phase of both walking and running, there is a requirement for muscles to generate an extension and abduction moment at the hip at the same time as an abduction moment at the knee, to resist joint motion in the opposite directions [47, 48]. Can GMAX serve both of these purposes simultaneously? Equally, can TFL contribute to generating abduction moments at both the hip and the knee in later stance? Both muscles serve to generate mechanical functions at the hip and the knee, with any force transmitted through the ITB also presumably creating a medially directed force on the lateral aspect of the knee. It is currently difficult to determine the timing or magnitude of forces that act through the ITB during tasks like walking or running as a result of TFL or GMAX contraction because of the complex interplay between the muscles and their activation. The secondary effects muscle forces have on factors like strain of the ITB or compression of the band against the knee also warrant investigation, because it is possible that changes in muscle forces may reduce the likelihood of injury. These factors are discussed in detail below (see Sects. 4.3 and 4.4).

### Knee Stability

The insertions of GMAX and TFL into the ITB have caused a number of researchers to speculate on the role these muscles, and, in turn, the ITB may have on lateral knee stabilization [1, 4, 14, 15, 17, 20, 21, 36, 44]. The literature supports a theory that the knee is indirectly stabilized as a byproduct of the muscles tensioning the ITB, though variation in the interpretation of the ITB’s distal attachments has produced a large number of conclusions on the exact role these attachments play in stabilizing the knee in the frontal plane [1, 4, 44]. For example, attachments to the patella stabilize the patella against medial dislocation [5, 15, 19, 20, 41, 49]. ITB attachments to the anterior and lateral tibia resist anterolateral subluxation of the tibia relative to the femur, playing an important redundancy role in the pivot shift mechanism of an anterior cruciate ligament-deficient knee [17, 42, 50]. However, all potential knee stabilisation mechanisms for the ITB are dependent on attachment location, loading, mechanical behaviour and posture.

Cadaveric studies have suggested ITB tension can induce lateral patella displacement and external tibial rotation [49]. It is presently difficult to assess whether passive forces generated in the ITB would be sufficient to counteract other muscular and external forces acting on the knee during movement. Cadaveric work has also helped to establish the ITB’s role in lateral knee stabilisation, in concert with the fibular collateral ligament, biceps femoris muscle and popliteus tendon [17]. Passive stability is maintained providing at least two of these structures are intact, although this may be posture dependent [1, 17, 20, 21]. Matsumoto and Seedhom [51] further contend that fibres from the ITB attaching to the femur also contribute to knee lateral stability. In a fully extended knee, the ITB insertions do nothing to prevent the anterior dislocation of an anterior cruciate ligament (ACL)-deficient knee [15]. However, once the knee is flexed past 30°, the ITB attachments are able to reduce the anterior translation of the tibia in an ACL-deficient knee (i.e., the pivot shift mechanism) [52].

A key shortcoming of the research cited above is that it is primarily based on cadaveric studies, where the forces applied through the ITB are purely passive, and it therefore ignores the potentially large forces transmitted from the GMAX or TFL during real-world activities like walking and running. As such, it is difficult to determine and precisely understand how the ITB contributes to stability of the knee without knowing how forces are applied in different postures. Furthermore, the role GMAX and TFL play in both knee and hip stability is also influenced by the role of both agonists and antagonists that may also generate moments about these joints.

### Knee Compression Forces

**Fig. 6:**
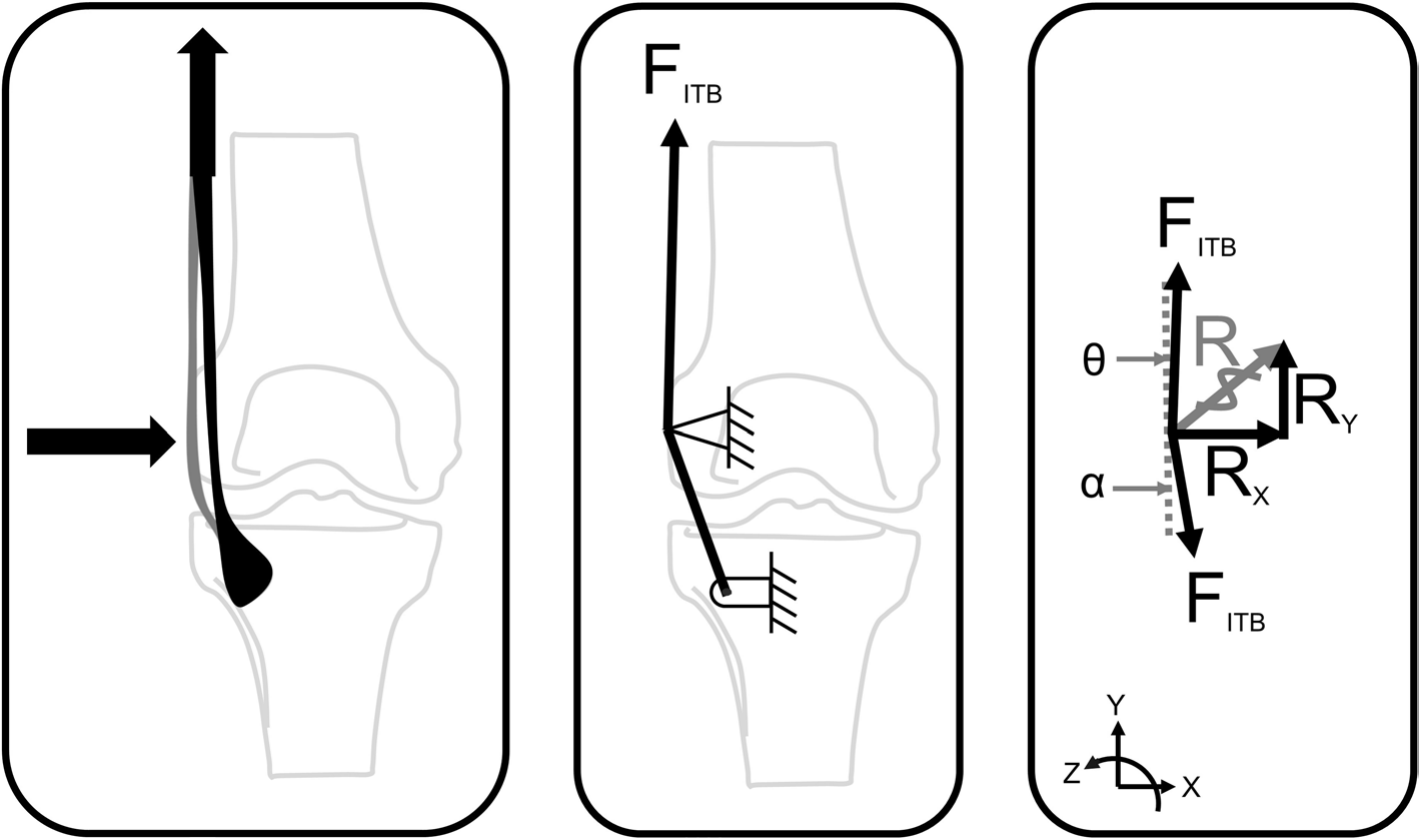
Free body diagram of a simplified analysis of the lateral knee compression forces caused by tensioning the iliotibial band (ITB). As the tension (*F*_ITB_) is increased, the lateral femoral epicondyle will experience a greater compressive force (*R*). Therefore, the lateral compression force (*R*_*x*_) could be represented as a function of the tension in the ITB (*F*_ITB_)

Stability of the knee joint may be achieved through the ITB’s resistance to external adduction moments, or also through the knee compression force applied to the femur through tensioning of the ITB. To further our mechanical understanding of the lateral compression of the ITB, we consider a simplified free body diagram (Fig. 6).

Magnetic resonance images reveal that the tissues between the distal ITB and the lateral femoral epicondyle undergo compression as the ITB is tensioned (Fig. 6, *R*) [5]. Therefore, the lateral compression force (*R*_*x*_) could be represented as a function of the tension in the ITB (*F*_ITB_) [26]. This lateral compression force would be significantly lower than the tension in the ITB, but would naturally increase with tension. This simplified analysis ignores the attachment and possible force transmission of the ITB to any other locations aside from Gerdy’s tubercle. Quantifying this relationship, especially as it relates to individual muscular contribution, will give us a clearer idea regarding the aetiology of pathologies like ITBS. Going one step further, considering the current theories for the aetiology of ITBS as a compression syndrome [5, 26], it closely follows that this added stability to the knee joint could create unfavourable excessive compression, particularly in the presence of varus knee torques.

## Ex vivo and In vivo Material Properties and Elastic Function

### Material Properties

It is important to understand the material behaviour of the ITB as a precursor to understanding its roles within locomotion and stability in the human body. The ITB facilitates the attachment of muscle (GMAX and TFL) to bone (pelvis, femur and tibia), and therefore it is likely to have tendon-like material properties, relevant for providing joint stability, but also potentially contributing to elastic energy storage and return, like the Achilles tendon [1, 10, 11, 36, 53]. Understanding the effect that ITB tension or joint posture has on the ITB’s stiffness in vivo is important to gauge the role that the ITB plays in the body. Furthermore, it is unknown how interventions, such as surgical releases [54] or surgical grafts (e.g., ACL reconstruction), might impact these material properties or force transmission pathways [55].

Ex vivo uniaxial testing of dissected ITBs has provided some indication of the elastic properties, summarized in Table 1 [10–12, 56]. These studies show that the ITB has an approximate Young’s modulus of 0.4 GPa, significantly lower than that of the Achilles tendon (~ 0.8 GPa) [57], and therefore the ITB is comprised of less stiff material.

**Table 1:** Summary of reported elastic moduli of the iliotibial band (ITB) measured ex vivo

Scenario	Citation	Elastic modulus [GPa]	Main findings
Ex vivo (lateral mid ITB)	[56]	0.397 ± 0.152	Significant influence of chemical fixation on elastic modulus
Ex vivo	[11]	0.392 ± 0.189	The region of ITB specimen did not affect thickness or stiffness within the mid-section of the ITB
Ex vivo	[12]	0.084 ± 0.030 (young) 0.369 ± 0.191(old)	Elastic modulus is significantly lower (less stiff) in younger donors than in older ones
Ex vivo	[10]	0.398 ± 0.017	Significant differences were found between the material properties of tendons and fasciae lata

While the general material properties of the ITB are well described, the mechanical behaviour of the whole structure is less clear. For instance, tensioning of the fibres of the ITB from anterior to posterior as the hip is extended means that different regions of the band are tensioned depending on the movement pattern [8]. Given the in-series musculature, the force transmission within the band is likely varied depending on which muscle is generating the forces, which in turn depends on the posture of the leg and the requirement for generating forces at any given point in time. Presently, little is known about how force transmission occurs as a result of the contraction of GMAX and/or TFL and how this impacts the structure of the ITB; for example, are there thickness differences between the anterior and posterior ITB fibres as a result of the forces generated by the GMAX versus TFL? Understanding how the ITB behaves in vivo requires the ability to directly measure the mechanical behaviour of the tissue during movement or muscle contraction.

Tateuchi et al. used shear-wave elastography to investigate the relationship between static single leg posture and the shear modulus of the ITB [7, 58]. The shear modulus is considered to be related to the stiffness of the tissue [59], but is also used as a surrogate measure of tension, since stiffness increases concurrently with tension [60]. Tateuchi et al. found that major hip adduction increased the shear modulus (16.9 kPa), while minor hip abduction significantly decreased shear modulus (9.5 kPa) [7]. This is an important initial finding in characterizing the stiffness of the ITB in vivo, and in understanding how static posture may influence stiffness or tension. While these data suggest more tension in the ITB in hip adduction, it is difficult to ascribe this to passive tensioning from stretch at the joint versus individual contribution from the activation of GMAX or TFL with ab/adduction. Given the paucity of information regarding when and how tension or stress is applied to the ITB, it would seem pertinent to apply advanced imaging techniques like shear-wave tensiometry [61] to further understand the function of the ITB and in-series musculature.

### Elastic Function

Human legs have spring-like tendons that allow for economic storage and release of energy during locomotion [62–65]. The Achilles tendon, plantar fascia, ITB and peroneus longus are all energy-saving structures [53], though the Achilles tendon is clearly the most dominant, contributing approximately 35–40% (35 J) of the positive work during the stance phase of running [64]. Given the ITB’s size, relative compliance and its similarity to other spring-like tendons that have important energy saving contributions, it is plausible that it absorbs and dissipates energy in a similar fashion to the Achilles tendon or plantar fascia.

Eng et al. used a sophisticated neuromuscular model to explore the potential role of the ITB to store energy [8]. Their model combined published EMG recordings of lower extremity musculature and kinematics of the joints to estimate the forces in the ITB during running [8]. Their findings suggested that the ITB stores up to 5% of the total positive work during a moderately paced run [8], which is approximately 14% of the work that the Achilles contributes [8, 53, 66]. Eng et al.’s model predicted that the posterior ITB (modelled as the portion that the GMAX muscle inserts into) is able to transmit larger forces than the anterior (modelled as the portion that TFL muscle inserts into), which would lead to a larger energy absorption [8]. It is likely that variability in the proportion of GMAX fibres that directly insert into the ITB would lead to variability in the energetic contribution of the ITB to whole body energetics; however, this is as yet untested. While there is little way of validating the findings of the model without direct measures of the stresses and strains experienced by the tendon, it provides an interesting insight into a mechanical function of the ITB.

Measuring the energetic contribution of soft tissues is difficult, due to the large number of degrees of freedom and the challenges with directly accessing their kinetic contributions to motion. Understanding the contribution of energy storage and return to individual tissues like the ITB requires new, accurate measures of the forces and/or the strains experienced by these tissues in vivo during dynamic activities, which presently do not exist. At the same time, we must also consider the two muscles (GMAX and TFL) that insert directly into the ITB and their potential for energetic modulation. While Kaplan contended that the small size of TFL would limit its potential for significant power generation, the large size of GMAX would, in addition to TFL, suggest reasonable ability to generate power and or store energy within the ITB [1]. Quantifying the force transmission within the ITB during dynamic activities using modern technologies (e.g., shear-wave tensiometry [61]) is essential to allow us to determine the ITB’s effect on the production of joint power.

## Clinical Significance

### Pathomechanics of Iliotibial Band Syndrome

ITBS is an overuse injury that can be debilitating for athletes [3, 25, 54, 67]. Pain at the lateral knee is commonly exacerbated by tensioning the band (e.g., single leg stance) [54]. ITBS is the most prevalent relative overuse injury at the lateral knee and can total 12% of all running-related injuries, as well as making a significant contribution to cycling and military-related injuries [54, 67–69]. The aetiology of ITBS has been a source of conflict in the literature, with a range of diagnostic and treatment strategies that address conflicting mechanisms or which are not well supported by rigorous clinical trials [44, 54, 68, 70, 71]. A lack of fundamental understanding of force transmission within the ITB, and therefore the stresses and strains experienced, underpins the lack of evidence and rationale for many ITBS treatments.

**Fig. 7:**
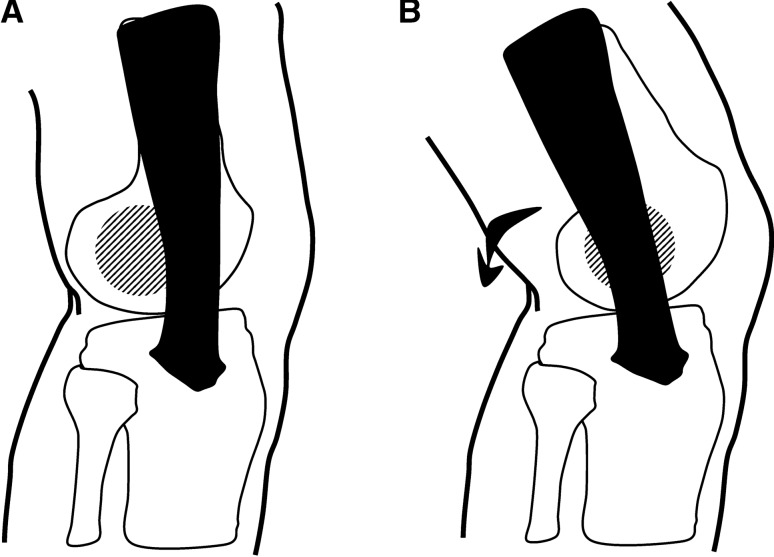
Iliotibial band (ITB) friction syndrome: **A** at full extension, the ITB (black) lies anterior to the lateral femoral epicondyle (lined region). **B** As the leg is flexed past ~ 30°, the ITB has been proposed to transverse the lateral femoral epicondyle as it moves posteriorly

There are two proposed mechanisms to describe how the mechanical behaviour and function of the ITB contribute to the aetiology of ITBS. Historically, ITBS was considered a friction injury [71]; the ITB traverses from anterior to the lateral femoral epicondyle in full knee extension (Fig. 7A), to posterior to the lateral femoral epicondyle as the knee is flexed beyond 30° (Fig. 7B) [3, 25, 54, 68, 70–72]. It was believed that the cyclic loading during activities like running and cycling caused the band to repeatedly traverse the lateral epicondyle causing irritation (“friction”) of the innervated fatty tissue underneath it [3, 25, 54, 68, 70–72].

**Fig. 8:**
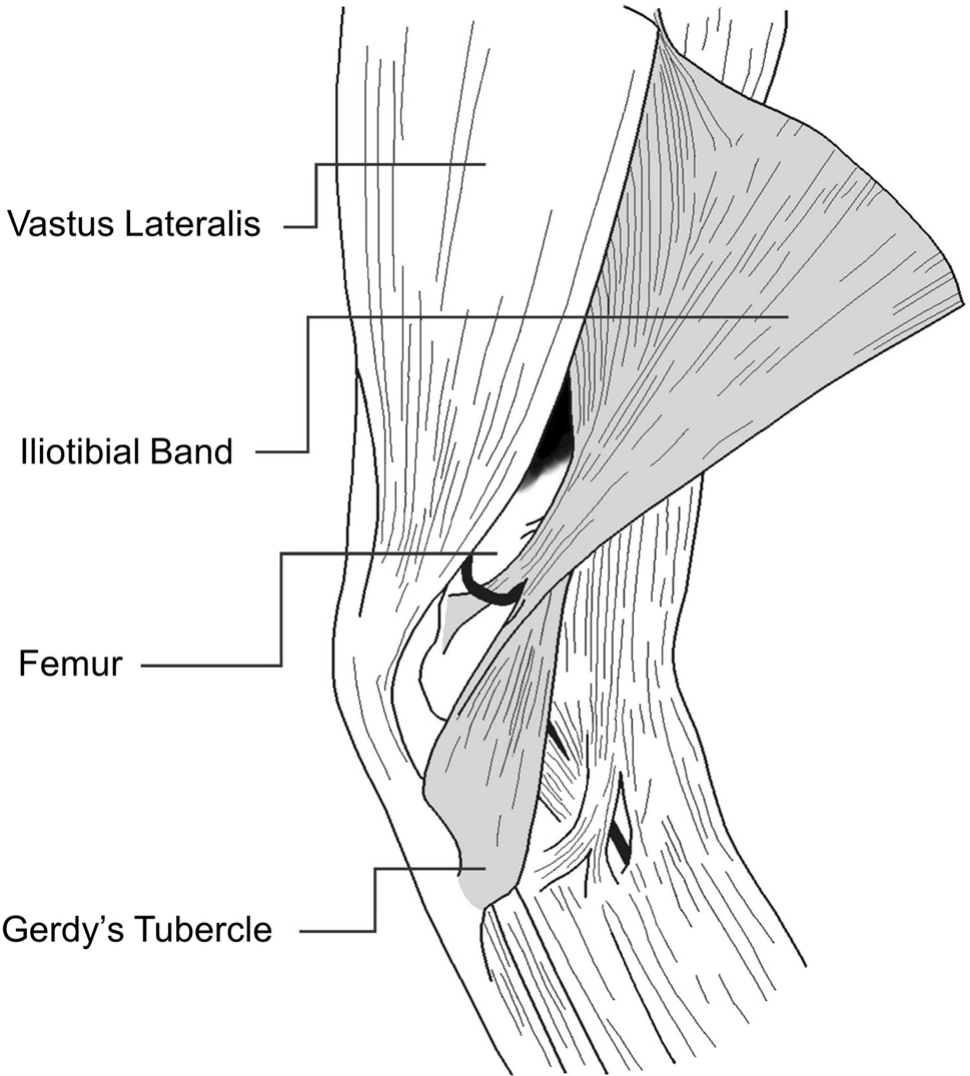
Early literature supported the tethering of the iliotibial band to the femur for all but the most distal end

More recently, the proposed friction mechanism of ITBS has been questioned [5, 26]. Contesting this view, Fairclough et al. first explained that the friction theory is an illusion created by the sequential load shifting of the fibres of the ITB from anterior to posterior as the band is tensioned [5]. In fact, the ITB is tethered to the distal femur, except for the upper portion of the lateral femoral condyle (Fig. 8) [1, 5], thus preventing the previously proposed traversing of the ITB over the lateral femoral condyle.

Therefore, it seems impossible that ITBS is induced by a friction mechanism. A new theory developed with the help of magnetic resonance imaging (MRI) showed that when the knee is flexed past 30°, the band compresses medially against the lateral femoral epicondyle as tension increases in the posterior fibres of the ITB (Fig. 6, left pane) [5, 26]. This compression is postulated to cause irritation of the highly innervated fat between the band and the bone, strongly suggesting that ITBS should be classified as a compression syndrome [26, 54, 67, 68].

### Running Kinematics

There have been many biomechanical studies investigating ITBS, outlined in a number of recent reviews [22, 23, 25, 73, 74], often creating more questions than answers. Is ITBS a function of lower limb kinematics as well as ITB force transmission? Are changes in the kinematics of the lower limbs the cause of ITBS or are they a compensatory strategy to accommodate and minimize pain? When investigating sagittal plane kinematics, Orchard et al. found no differences between participants’ healthy (asymptomatic) and affected limbs [37]. Prospectively, Noehren et al. [6] and Friede et al. [30] found that individuals who would go on to develop ITBS exhibited greater hip adduction and knee internal rotation during running when compared with matched controls. Foch et al. found that runners with past histories of ITBS had reduced hip adduction when compared to healthy controls [75, 76]. Noehren et al. found that male runners with ITBS ran with significantly greater hip internal rotation and knee adduction angle [77]. These altered kinematics combined with the increased prevalence of ITBS in men (50–81% of those affected) [22] may allude to pain compensation strategies resulting in kinematic changes. This is supported by studies showing that there is a progressive reduction in peak hip adduction angle during prolonged runs, which may be associated with strategies to reduce pain [6, 29, 30, 75, 76, 78, 79]. Overall, these papers suggest that runners may adopt a pattern that places less strain on the ITB once pain is present.

### Diagnosis of ITBS

Diagnosis of ITBS relies on clinical reasoning and is primarily a diagnosis of exclusion, suggesting that enhanced diagnostic criteria are needed. Generally, runners with ITBS report lateral knee pain approximately 2–3 cm proximal to the lateral tibiofemoral joint line in the region of the lateral femoral condyle. The onset of pain is often insidious in nature, preceded by a recent spike in running loads, usually consisting of an increased running distance, or increased volume of downhill running [72, 80]. Individuals with particularly irritable ITBS may report pain reproduction in their stance limb during stair descent as hip extension is coupled with knee flexion as the TFL musculature contracts eccentrically to assist in lower limb control [81]. Running, particularly downhill or fast running, may also exacerbate ITBS [81]. Other sources of lateral knee pain should be ruled out, including patellofemoral pain (exacerbated by deep squats, prolonged sitting and stair descent), lateral meniscal lesions (joint line pain with a history of a memorable event, such as twisting on a loaded knee), lateral synovial plica syndrome (screened via palpation of the lateral plica band), and a distal femoral bone stress injury (screened with the Fulcrum test, which has an excellent negative likelihood ratio of − 0.09, but should be confirmed with MRI if a bone stress injury is suspected) [82–85]. Gluteal tendinopathy and lumbar radiculopathy commonly refer pain to the lateral thigh and knee and should also be ruled out in patients presenting with suspected ITBS [86]. Imaging is not particularly helpful in diagnosing ITBS [70].

**Fig. 9:**
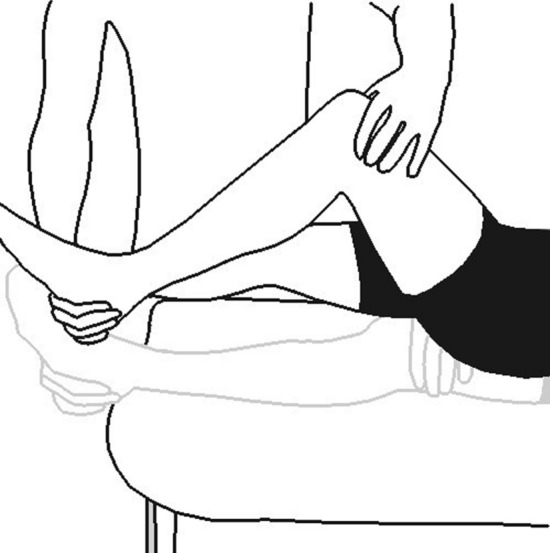
For the Noble compression test the patient starts by lying supine on the examination table. The examiner applies pressure on the lateral epicondyle of the femur as they extend the patient’s leg

The Noble compression test is the sole diagnostic test used in clinical examination of the runner with suspected ITBS. Briefly, the Noble compression test is conducted by applying manual pressure to the patient’s lateral knee, 1–2 cm proximal to the lateral femoral condyle as the knee is passively extended through a range of motion from 60° through full extension (Fig. 9). Reproduction of lateral knee pain as the knee is in a position of approximately 30° of knee flexion is considered a positive Noble compression test [68]. Importantly, the Noble compression test has unknown positive and negative likelihood ratios, suggesting that caution should be used when interpreting the test results and calling into question this test’s clinical utility.

**Fig. 10:**
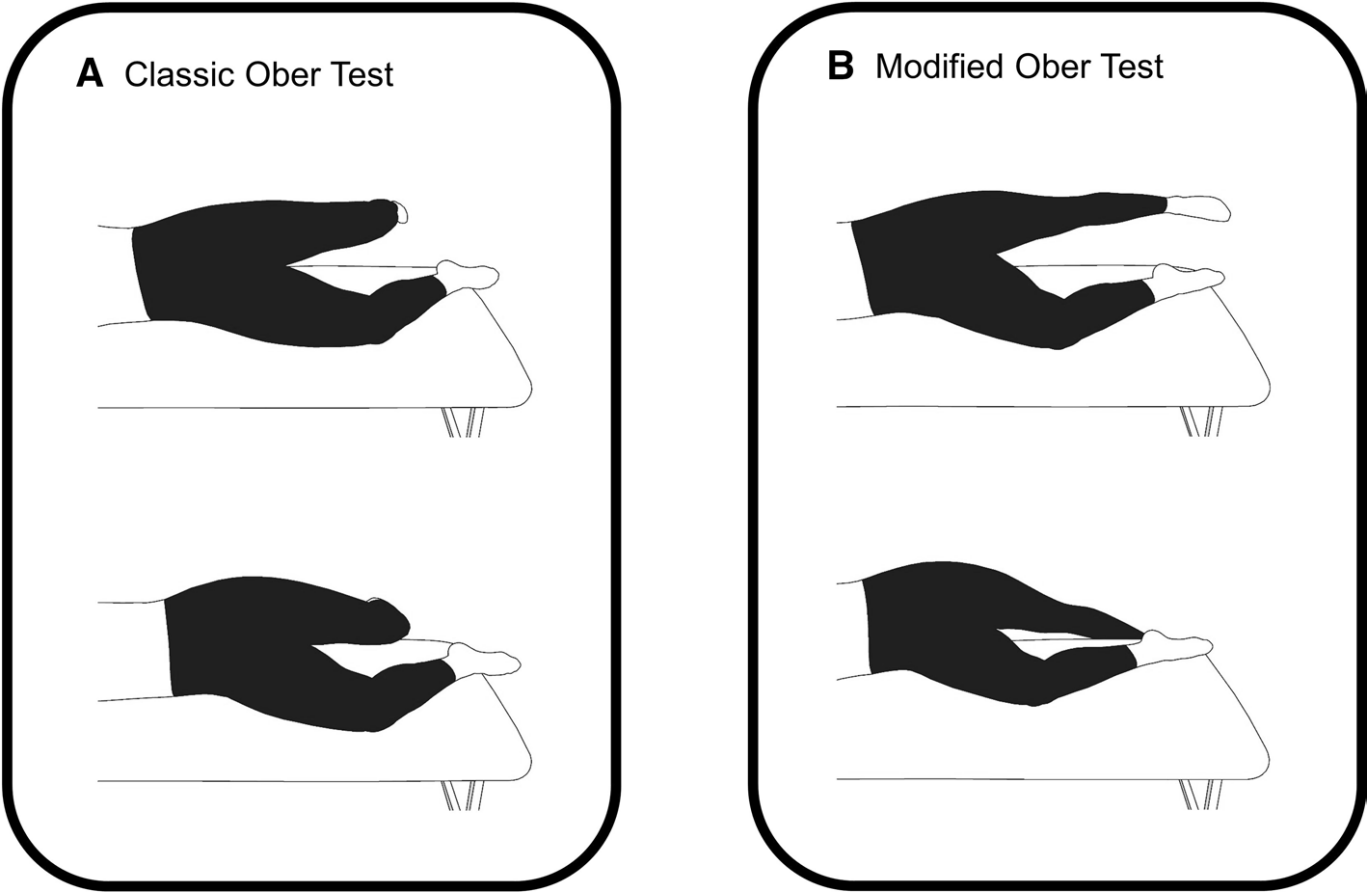
**A** The classic Ober test flexes the knee to 90°, extends the hip, and adducts the leg. **B** The modified Ober test keeps the knee fully extended, extends the hip, and then adducts the hip down

While not diagnostic tests, the classic and modified Ober’s tests are commonly used by clinicians to assess ITB “tightness” (Fig. 10) [87–89]. For the classic Ober’s test, the patient lies on their side with their hips at neutral flexion [87]. An examiner flexes the patient’s affected knee passively to 90° while adducting and extending the hip posterior to the unaffected leg, which is kept in line with the trunk [87, 89]. The examiner then allows gravity to further adduct the leg lower while limiting transverse and sagittal plane movement [89]. A positive Ober is indicated by inability to further adduct under gravity past the horizontal [87, 89]. Similarly, the modified Ober’s test is identical except that the knee remains fully extended and the pelvis is manually stabilized [32, 89]. The rationale for introducing a modified Ober’s test was to reduce any possibility of influence from a tight rectus femoris, limiting the test’s findings [89].

Both versions of the Ober test rely on the assumption that an injured ITB is tighter than a healthy ITB; however, neither the classic nor the modified Ober’s test appear to actually assess ITB “tightness”. In fact, neither version of the test was affected when the ITB was transected in a cadaveric investigation [89]. Rather, a positive Ober test of either version was attributed to restrictions of the hip capsule and gluteus medius and minimus musculature [89]. Clearly, neither version of the Ober’s test is helpful in either diagnosing ITBS or assessing ITB “tightness”. A positive test in the individual with ITBS may be a spurious finding or may be present due to patient guarding in the presence or fear of pain reproduction during the physical examination.

Individuals with ITBS often present with hip abductor weakness, yet hip abductor weakness, curiously, is not a risk factor for ITBS [22]. The review from Mucha et al. [78] provides some evidence of strength deficits in runners with ITBS, but all of this evidence is from cross-sectional studies. No prospective study that we are currently aware of has assessed this (which is required to provide causative evidence). As such, the same conclusion should be drawn as in the 2012 review [22], which is that “hip abductor weakness can be considered a result of ITBS rather than a cause of ITBS”. Based on the lack of prospective studies and no clear relationship between hip abduction strength and ITBS, we believe that pain caused by the distal compression of the highly innervated tissues deep in the ITB in runners with ITBS may inhibit the proximal hip musculature, namely the TFL and gluteus maximus, in an adaptive strategy to reduce tension in the ITB. Thus, hip weakness is a concomitant finding that accompanies ITBS rather than a causative factor of this injury.

### Treatment of ITBS

The scientific literature on the treatment of individuals with ITBS is generally of low quality, consisting of either narrative reviews or case series. Despite the relatively low quality of the literature, progressive overload and graded exposure to increasingly challenging activities are consistently described as integral to the recovery of individuals with ITBS. Clinicians wishing to implement treatment programs described in the literature are hampered by poorly described therapeutic exercise programs, lacking key details such as exercise intensity (e.g., percent of 1-RM), repetitions and frequency [72, 81, 90]. The lack of rigorous study designs evaluating treatments for ITBS further hinders clinicians’ ability to determine the necessary components of rehabilitation programs. The shortcomings in understanding ITB function and ITBS become clear when we consider the treatment options for ITBS described in the literature. Foam rolling, ITB “stretching” and hip strengthening are mainstays in the treatment of individuals with ITBS [72, 81]. Yet, the basis of these treatments is not supported by our current understanding of the structure and function of the ITB.

Foam rolling of the ITB is commonly prescribed for runners with either ITB “tightness” and/or ITBS [72]. Changes in flexibility that result from foam rolling are short-lived or insignificant, and any pain relief resulting from foam rolling is only temporary in nature, lasting as little as a few minutes [91, 92]. Since ITBS is considered a compression syndrome [26], prescribing additional compression, via foam rolling, lacks biological and mechanical justification, and may conceivably exacerbate ITBS.

Stretching of the ITB is similarly problematic considering that the assessment of ITB “tightness”, i.e., the Ober test, that is used to determine if an individual may benefit from stretching the ITB, is not a valid assessment of ITB tightness [89]. A recent study by Friede et al. investigated the stiffness of the ITB through shear-wave elastography in healthy participants and those with ITBS symptoms after a 6-week training period [30]. This training period aimed at first reducing the lateral knee pain before strengthening the hip abductor and external rotator muscles. This study found no differences in ITB stiffness between healthy participants and those with ITBS [30]. This finding further challenges the notion that increased stiffness of the ITB causes ITBS pain. Furthermore, an increase in ITB stiffness may be a sign of resolution of ITBS rather than a cause of ITBS. After a 6-week intervention, individuals with ITBS exhibited *an increase* in ITB stiffness and an absence of pain or other ITBS-related symptoms [30].

Hip strengthening is also commonly prescribed for individuals with ITBS. While hip weakness and pain resolved concurrently in runners with ITBS enrolled in a hip-strengthening program [90], caution is urged concerning attaching causation to hip weakness considering that hip weakness follows the onset of ITBS [22]. A biomechanical rationale for prescribing hip strengthening for ITBS is similarly lacking, even though greater hip adduction is thought to increase ITB strain [93, 94]. First, hip strength does not appear to be related to hip adduction during running [95, 96], despite this being a widely held belief among clinicians. Secondly, hip strengthening does not result in reduced hip adduction during running [97]. It is possible that intensive hip strengthening results in enhanced tissue qualities of the ITB and related structures, via mechanotherapy [30, 98]. Another possible reason that hip strengthening results in pain relief in individuals with ITBS is that any targeted loading exercise, such as intensive hip strengthening, can alter central pain processing and reduce local hyperalgesia [99].

Interventions that directly target running biomechanics suspected to increase ITB strain, and subsequent compressive loads acting on the lateral knee, may have promise. For instance, Meardon et al. used a subject-specific musculoskeletal model during running to show that a wider step width reduces ITB strain [100]. Feedback on step width can easily be provided with a full-length mirror during treadmill running. Running with a higher cadence (stride frequency) reduces ITB strain and strain rate in a similar musculoskeletal model [101]. Cueing an increase in running cadence can easily be accomplished during routine, in-field runs through the use of commercial wearable devices [102]. However, rigorous study of biomechanical interventions, via randomized controlled trials, for the treatment of individuals with ITBS is currently lacking.

Despite substantial research on this topic, even the most recent work challenges our commonly accepted and widely practiced standards for diagnosing and treating ITBS. Though we are having success in treating patients’ concerns and pain, the effect of our actions on the ITB remains a mystery. Current approaches to diagnosis, treatment and understanding of ITBS function are insufficient, indicating a much larger issue of a lack of understanding of the ITB’s fundamental mechanical function. Further, this research is primarily dependent on simplified musculoskeletal models that often fail to include the GMAX’s potential contribution to ITB strain [100, 101].

## Conclusion

Our article discusses the important topic of the discrepancies in the described function of the ITB and the controversies associated with the diagnostic and treatment strategies applied for this condition. In this review, we have synthesized the anatomy and biomechanics of the ITB and related muscles to propose novel ideas about how stress is created in the iliotibial band, and how this might relate to debilitating pathologies like ITBS. We feel that the complex relationship between ITBS and mechanical function of the in-series hip musculature is a promising area for future work to understand why some athletes develop ITBS when others do not, and how best to treat patients clinically.
